# New Insights Into the Pathogenesis of Alzheimer's Disease

**DOI:** 10.3389/fneur.2019.01312

**Published:** 2020-01-10

**Authors:** Liyuan Fan, Chengyuan Mao, Xinchao Hu, Shuo Zhang, Zhihua Yang, Zhengwei Hu, Huifang Sun, Yu Fan, Yali Dong, Jing Yang, Changhe Shi, Yuming Xu

**Affiliations:** ^1^Department of Neurology, The First Affiliated Hospital of Zhengzhou University, Zhengzhou University, Zhengzhou, China; ^2^Academy of Medical Sciences, Zhengzhou University, Zhengzhou, China

**Keywords:** Alzheimer's disease, amyloid, tauopathies, gamma rhythm, prions, ghrelin, pericytes, infection

## Abstract

Alzheimer's disease (AD), a common neurodegenerative disease in the elderly and the most prevalent cause of dementia, is characterized by progressive cognitive impairment. The prevalence of AD continues to increase worldwide, becoming a great healthcare challenge of the twenty-first century. In the more than 110 years since AD was discovered, many related pathogenic mechanisms have been proposed, and the most recognized hypotheses are the amyloid and tau hypotheses. However, almost all clinical trials targeting these mechanisms have not identified any effective methods to treat AD. Scientists are gradually moving away from the simple assumption, as proposed in the original amyloid hypothesis, to new theories of pathogenesis, including gamma oscillations, prion transmission, cerebral vasoconstriction, growth hormone secretagogue receptor 1α (GHSR1α)-mediated mechanism, and infection. To place these findings in context, we first reviewed the neuropathology of AD and further discussed new insights in the pathogenesis of AD.

## Introduction

Alzheimer's disease (AD), which was first described by German Bavarian psychiatrist and neurologist Alois Alzheimer in 1907 (1), is the most common degenerative central nervous system disease in the elderly. According to the Alzheimer's Association, AD accounts for an estimated 60–80% of dementia cases (2). At present, there are 50 million AD patients worldwide, and its incidence doubles every 5 years after the age of 65 years (3). The main clinical manifestations are cognitive dysfunction, memory loss, and abnormal changes in personality. The pathological cause of AD is considered to be the senile plaque (SP) formed by amyloid beta (Aβ) and neurofibrillary tangles (NFTs) composed of phosphorylated tau protein, in the hippocampus.

AD can be late onset (LOAD) and sporadic (SAD) or early-onset (EOAD) and familial (FAD) (4). FAD is mainly associated with mutations in the Aβ precursor protein (*APP*) and presenilin genes *PSEN1* and *PSEN2*, whereas SAD has a complex etiology, involving genetic, environmental, metabolic, viral, and other factors (5). Apolipoprotein E (APOE) is a polymorphic protein with three isoforms, APOE2, APOE3, and APOE4, where *APOE4* is the strongest genetic risk factor for SAD (6, 7). According to statistics, 40–50% of EOAD and 80% of LOAD are associated with *APOE*. The strong affinity of APOE for Aβ affects the production, hydrolysis, and elimination of Aβ (8–10). Endogenous expression of APOE4 in stem-cell-derived neurons promotes the release of phosphorylated tau and predisposes neurons to injury and calcium dysregulation. Interestingly, *APOE2* is a protective factor that reduces the incidence of AD and Aβ accumulation and delays the age of onset (11–13). Some researchers have found that APOE4 expression in mouse models increases oligomer expression and plaque deposition, whereas this is reversed in expression of APOE2 (14). However, there is clear *in vivo* evidence that both APOE2 and APOE4 isoforms are involved in the process of Aβ aggregation and deposition and associated with neurodegeneration, although the effect of APOE4 appears to be much stronger than that of APOE2 (15). Regardless, small-molecule inhibitors of APOE/Aβ interaction may provide a therapeutic option for SAD, which accounts for more than 95% of AD.

There are many hypotheses to explain AD pathogenesis, involving the amyloid cascade (16), tau hyperphosphorylation (17), neurotransmitters, and oxidative stress (18). However, the underlying causes and optimal treatment plans are still elusive. At present, there are a few drugs available that improve symptoms, mostly targeting Aβ and tau, but these cannot delay progression of the disease. Researchers are beginning to explore new theories of the pathogenesis of AD from different perspectives, such as gamma oscillations, prion transmission, cerebral vasoconstriction, growth hormone secretagogue receptor 1α (GHSR1α)-mediated mechanism, and infection. Discoveries in these areas make it possible to reasonably explain the pathological mechanisms of AD and suggest potential effective treatments for AD. Herein, we review the two most recognized hypotheses and focus attention on novel developments in the pathophysiology of AD.

## Pathogenesis Hypotheses

Most of the recently proposed pathogenic mechanisms are derived from two fundamental hypotheses: the amyloid cascade hypothesis and the tau hyperphosphorylation hypothesis. First, we will review these two accepted hypotheses and the clinical research targeting them.

### Amyloid Cascade Hypothesis

**Figure 1:**
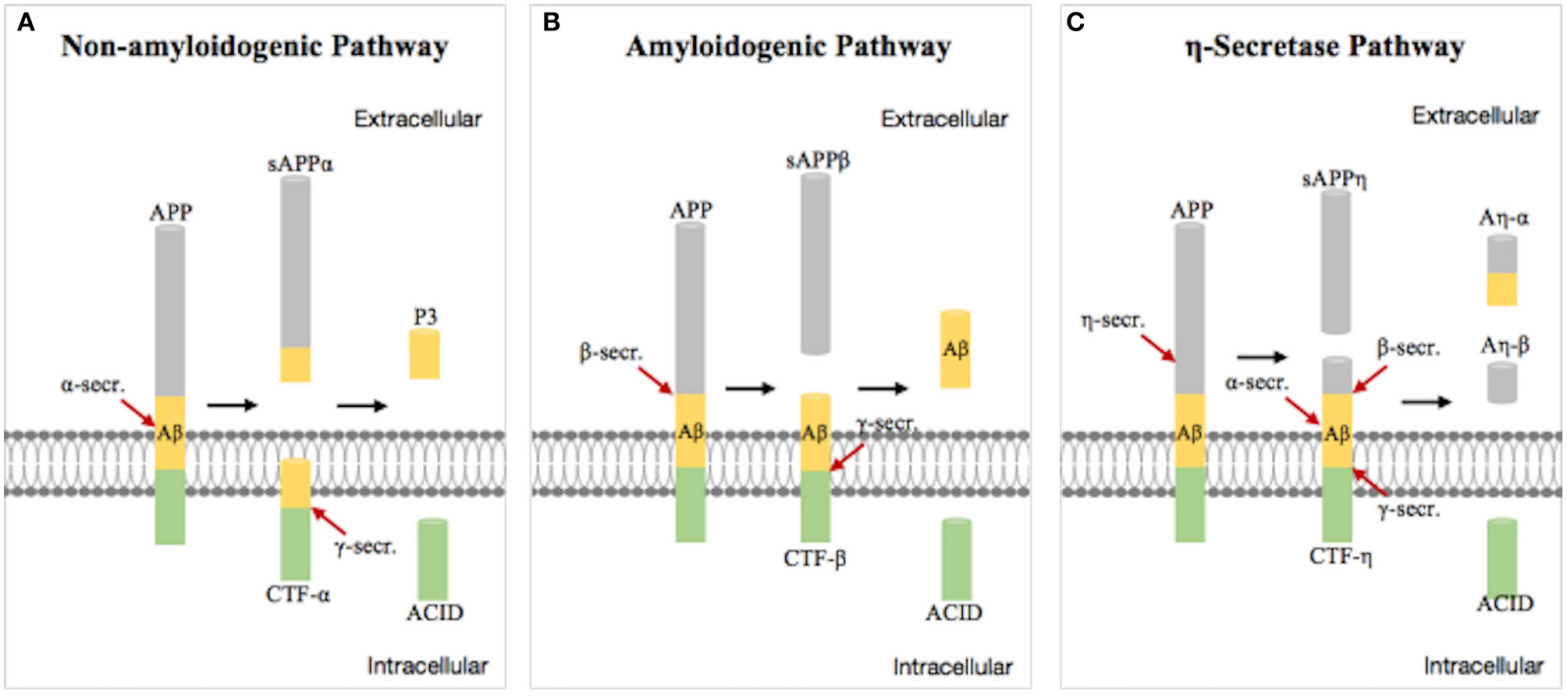
Schematic diagram of the progressive cleavages of the amyloid beta (Aβ) precursor protein (APP) transmembrane domain. Aβ peptide is generated from APP processing via the amyloidogenic pathway **(B)**. **(A,C)** are non-amyloidogenic pathological pathway under physiological conditions.

Aβ plaques were first proposed by Paul Blocq and George Mannesco when they discovered “circular accumulation in the brains of elderly patients” in 1892. After nearly a 100 years of research, Glenner isolated “beta-amyloid” from the meningeal vessels of Alzheimer cases and partially identified the peptide sequence (19). The amyloid hypothesis was first proposed by John Hardy and David Allsop in 1991 (20). Aβ is a transmembrane protein which is produced by hydrolysis of the Aβ precursor protein (APP) via the amyloidogenic pathway. Studies have shown that APP produces C-terminal fragments under the hydrolysis of α-, β-, γ-, and η-secretases by three pathways (21) (Figure 1). The first non-amyloidogenic pathological pathway produces products which are neurotrophic and neuroprotective for nerve cells, such as the C-terminal fragment (CTF)-α, the soluble ectodomain of APP-α (sAPPα), and other smaller fragments, through the involvement of α- and γ-secretases under normal circumstances. The second pathway is the amyloidogenic pathological pathway in which APP is cleaved to CTF-β by β-secretase and then different lengths of Aβ peptides by γ-secretase, including Aβ42 which is more prone to aggregation and plaque formation than Aβ40 and has stronger neurotoxicity (22, 23). The third pathway is the alternative processing route under physiological conditions by η-secretase.

Aβ in SPs is thought to be the initiating factor in the pathology of AD (24, 25). Aβ deposited in the hippocampus and basal segment, in the form of neurotoxic amyloid plaques, recruits more Aβ to form insoluble aggregates and induces mitochondrial damage (26), unstable homeostasis, and synaptic dysfunction (27). Microglia and astrocytes are activated and induce related inflammatory reactions and oxidation. Eventually, neuronal dysfunction and apoptosis occur, leading to AD. Tau protein kinase 1 can be activated by Aβ, leading to abnormal phosphorylation of tau protein and promoting the formation of paired helical filaments (PHFs) and NFT, which accelerate the development of tau pathology (28). Soluble Aβ oligomers are suggested to be more toxic than Aβ cellulose bodies (29). In 2011, Ferreira officially proposed the “Aβ oligomer pathogenic theory,” suggesting that soluble Aβ oligomers are the initiating factors leading to a series of pathological changes in AD (30). An increase of Aβ oligomers in the cerebrospinal fluid (CSF) of AD patients has been reported in many studies (31). Aβ oligomers begin to accumulate *in vivo* 10 years or even decades before clinical symptoms and contribute to long-term potentiation (LTP) inhibition as well as enhanced long-term depression (LTD) (32), by acting on multiple receptors including *N*-methyl-d-aspartate (NMDA)-type glutamate and α7-nicotinic acetylcholine (α7-nACh) receptors (33), leading to synaptic dysfunction and impaired learning and memory (34). Aβ42 oligomers also cause oxidative damage to synaptic membranes and induce hyperphosphorylation of tau protein (35, 36).

Currently, the goals of therapeutic strategies based on the Aβ hypothesis are to reduce Aβ formation and aggregation and increase Aβ clearance (16, 24, 37) (Table 1). The most direct action is to reduce Aβ production by controlling BACE1 and γ-secretase activity (38–40). However, γ-secretase inhibitors lack substrate specificity for APP and are toxic to many organs (41). The drug avagacestat, which was the first to undergo clinical trials, has serious side effects such as tumors, gastrointestinal reactions, and rashes and did not achieve the desired effects; as such, related trials have ceased (42–44). The use of Encore's tarenflurbil, which has good security, has also been terminated because there is no obvious improvement in cognitive dysfunction (45). Other drugs have been disappointing to date, in some cases performing worse than placebos, with increased adverse reactions (e.g., semagacestat) (44). By contrast, BACE1 inhibitors have higher substrate specificity and are one of the main areas for anti-AD drug development. However, many Phase III trials have not shown significant clinical benefits and showed unanticipated adverse side effects (46), such as the inhibitor verubecestat, although it can reduce Aβ in CSF by up to 90% (47, 48). The termination of related clinical trials was announced early in 2018. In July 2019, a Phase II/III trial of the BACE1 inhibitor umibecestat was also terminated due to cognitive deterioration in participants.

**Table 1:** Part of clinical studies on therapies in Alzheimer's disease (AD).

**Target**	**Drug**	**Clinicaltrials.gov**	**Study phase**	**Status**	**Main reasons for failure**
BACE1	Verubecestat (MK-8931)	NCT01953601	III	Terminated	Lack of efficacy
	Umibecestat (CNP520)	NCT03131453	II/III	Terminated	Toxicity
γ-Secretase	Avagacestat	NCT00890890	II	Terminated	Toxicity and lack of efficacy
	Semagacestat	NCT01035138	III	Completed	Toxicity and lack of efficacy
	Tarenflurbil (MPC-7869)	NCT00380276	III	Terminated	Lack of efficacy; increased adverse reactions; no clear reduction in amyloid beta (Aβ)
Aβ	Crenezumab	NCT02670083	III	Terminated	Lack of efficacy
	Aducanumab (BIIB037)	NCT02484547	III	Terminated	Lack of efficacy
	Azeliragon (TTP488)	NCT02080364	III	Terminated	Lack of efficacy
tau	LMTM (TRx0237)	NCT01626378	III	Completed	Toxicity and lack of efficacy
	Epothilone D	NCT01492374	I	Completed	Toxicity
	AADvac1	NCT02579252	II	Unknown	–

*Data sources: www.clinicaltrials.gov*.

*BACE1, β-site amyloid precursor protein cleaving enzyme 1; Aβ, amyloid beta; p-tau, hyperphosphorylated tau peptide*.

In addition, animal experiments have demonstrated that it is feasible to enhance the clearance and degradation of Aβ (49) or promote the delivery of Aβ to the periphery (50). Active immunity and passive immunity are research hot spots. This year, some Phase III trials, such as those for crenezumab and aducanumab, were terminated because they were ineffective and did not reach the primary endpoint, although they were able to reduce Aβ deposition.

Researchers have also attempted to reduce Aβ aggregation to improve brain pathology and cognition in mice (51), by using agents such as the endoglycosylation receptor inhibitor TTP488 (azeliragon) which showed good cognitive improvement in Phase II trials (52). However, the clinical Phase III trial was terminated because the desired effect was not achieved.

Recently, researchers at the University of Washington developed small synthetic alpha-peptides that target and inhibit small toxic oligomers and block Aβ aggregation at an early stage. Good results were observed in AD mouse disease models and *Caenorhabditis elegans* (a nematode model of AD). However, whether this technique can be applied to humans is yet to be studied (53).

### Tau Hyperphosphorylation Hypothesis

Tau is a microtubule-associated protein produced by alternative splicing of the *MAPT* gene (54). In 1988, Claude Wischik isolated tau from plaques in the brains of AD patients, demonstrating for the first time that tau protein may be the cause of dementia (55). Tau is mainly found in neuronal axons of the brain (56), combined with microtubules (MTs). The function of tau is to maintain microtubule structure and cytoplasmic transport function (57, 58), maintain synaptic structure and function (59), and regulate neuronal signaling. Tau is also a phosphoprotein whose phosphorylation and dephosphorylation may depend on the balance of protein kinase and protein phosphatase activity and is regulated by brain development. Under normal conditions, tau has few phosphorylation sites and negatively regulates the binding of tau to microtubules. Under pathological conditions, the phosphorylation of tau saturated.

The development of tau pathology is a complex multifactorial process. Hyperphosphorylated tau in AD patients' brains causes configuration changes and the loss of tubulin polymerization capacity (60, 61), resulting in defective microtubule functioning (62).

The elevated levels of cytosolic tau lead tau–tau interactions and polymerization to form insoluble PHFs and straight filaments (SFs) that result in the formation of intraneuronal fibrillar deposits known as NFTs (60). NFTs reduce the number of synapses, produce neurotoxicity (63), and cause cell dysfunction (64). Experiments have shown that hyperphosphorylation of tau is positively correlated with the degree of tau aggregation and the pathological severity of AD (65). Tau, rather than Aβ, determines cognitive status (66). In addition, the acetylation and truncation of tau inhibit its ability to bind to microtubules and also promotes tau aggregation, mitochondrial dysfunction, and synaptic deficits (67–69). Interestingly, p-tau has been shown to spread between cells (70). Small soluble tau may be more harmful than NFT which can not only help the spread of pathological tau but can also affect neurodegeneration and cognition (71). In summary, due to its complexity, the pathogenesis of pathological tau remains to be elucidated.

In recent years, tau has gained much attention, in part because of the failure of various Aβ-targeting treatments in clinical trials and because tau pathology correlates better with cognitive impairments than do Aβ lesions (Table 1). Inhibitors of kinases and tau aggregation, stabilizers of microtubules, and immunotherapeutic drugs have recently been investigated. Most of them show some toxicity and lack of efficacy, such as the inhibitor of tau aggregation, LMTM (TRx0237) (72). The antifungal molecule epothilone D proved to increase the number of microtubules, reduce the number of abnormal axons, and improve tau-related pathology in a mouse model of tau lesions (73, 74). However, the clinical trial of epothilone D was terminated because of adverse effects. AADvac1 as a tau vaccine showed good results in terms of safety and immune response in AD patients. However, further research is needed to demonstrate its clinical efficacy (75).

## New Insights Into the Pathogenesis of AD

With the prospect of an increasingly aging society, the number of AD patients and sociomedical burdens will increase dramatically. Currently, cholinesterase inhibitors (AChEIs) and the NMDA receptor antagonist are the only therapies for AD (76). However, these can only relieve symptoms and not delay the progress of AD (77–79). Moreover, three cholinesterase inhibitors, namely, donepezil, rivastigmine, and galantamine, which are approved by the US Food and Drug Administration, were proven to increase side effects, such as nausea, vomiting, and diarrhea. Although the NMDA receptor antagonist memantine showed good effects on improving cognitive function and behavioral disturbance scores (80), it causes severe hypotension, leading to fainting, and falls (81). According to statistics, AD drug development had a high failure rate of 99.6% in the decade between 2002 and 2012 (82). Based on these above unsatisfactory results, researchers are constantly proposing new pathogenic mechanisms.

### Gamma Oscillations Ameliorate Pathology and Cognitive Impairment in AD

Gamma oscillations are rhythmic fluctuations of brain waves caused by activation of local circuits of excitatory and fast-spiking inhibitory neurons in the local field potential (LFP), resonating at 20–50 Hz and are associated with numerous higher-order cognitive functions, such as memory formation and attentional selection (83). Changes in gamma oscillations were reported in a variety of neurological diseases including AD. In TgCRND8 transgenic mice, the θ-γ crossover frequency coupling in the hippocampus was impaired before plaque formation in the brain (84). Furthermore, the reduction of slow gamma power in CA1 of 3xTg mice can result in impaired memory function (85). Moreover, hippocampal oscillations were also observed to affect spatial memory in a mouse model of tau pathology (86). These pieces of evidence suggest that the reactivation of gamma oscillations may play a role in protecting cognitive function in AD.

**Figure 2:**
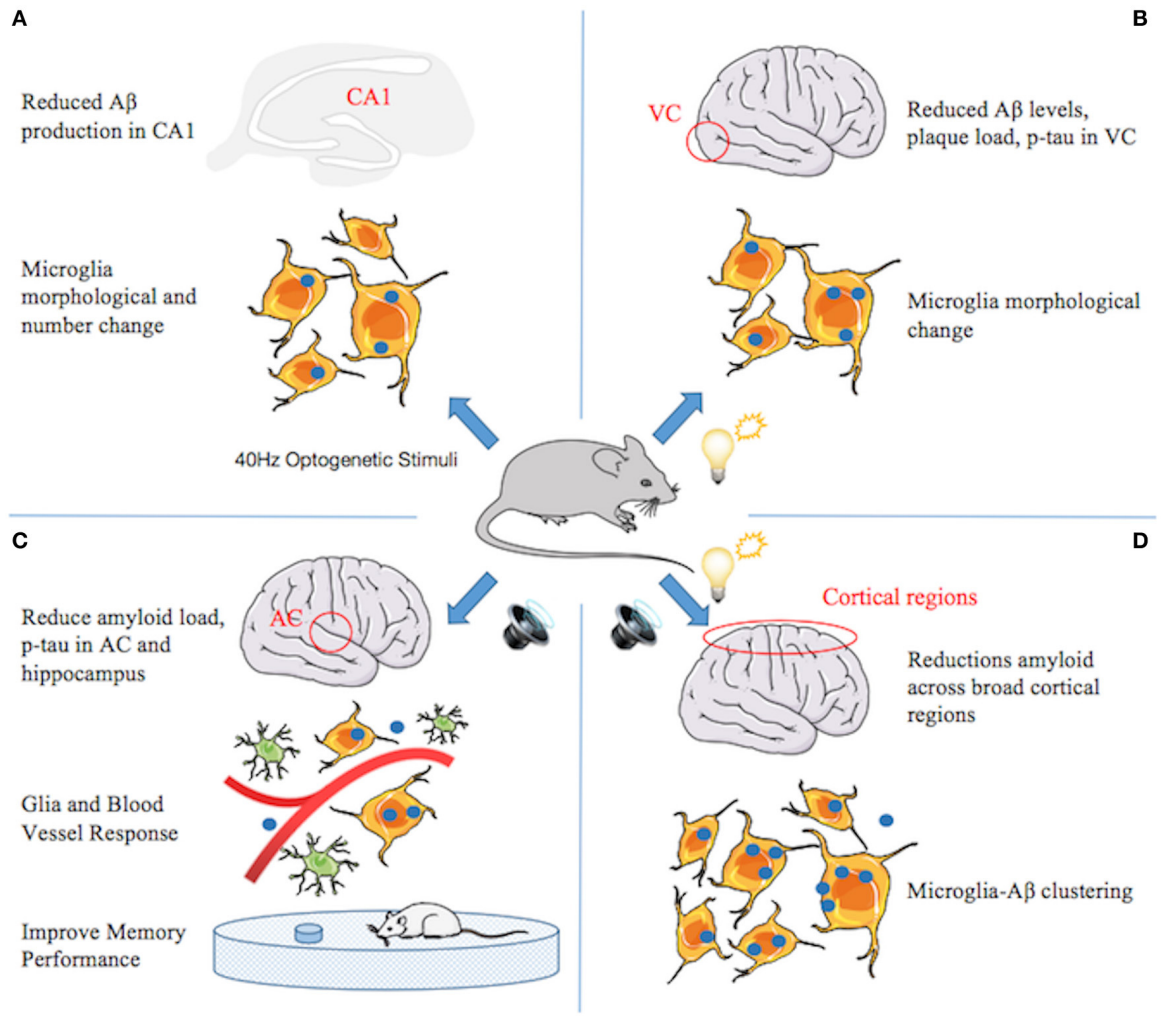
A series of studies from Li-Huei Tsai indicating that gamma stimulations ameliorate pathology and cognitive impairment in Alzheimer's disease (AD). **(A)** Gamma induced by optogenetic stimuli reduced amyloid beta (Aβ) production in CA1, increased number and cell body diameter of microglia, and reduced the process length of microglia compared with the control group, indicating an engulfing state of microglia. The percentage of microglia co-localized with Aβ in the cell body increased, suggesting that gamma stimulation triggers microglia to increase Aβ uptake. **(B)** Gamma induced by light flicker reduces Aβ levels and plaque load in the visual cortex (VC). Microglia changes similar to **(A)**, except that the number does not change. **(C)** In 5xFAD mice, 40-Hz auditory stimulation improves memory performance and reduces amyloid load and tau phosphorylation and seeding in the auditory cortex (AC) and hippocampus. The changes of microglia similar to **(A)** and the number of astrocytes increased. Furthermore, blood vessel diameter increased. **(D)** Combined auditory and visual stimulation induces a clustering phenotype response by microglia and reduces amyloid load across broad cortical regions.

Surprisingly, studies that replaced endogenous mouse *APOE4* with the AD-linked human *APOE4* gene showed that alleviated gamma impairments after replacement in mice rescued learning and memory deficits (87). Iaccarino and colleagues observed that behaviorally driven gamma oscillations in the AD mouse model are reduced before plaque formation or cognitive decline begins (88) (Figure 2). Using optogenetics in the hippocampus of 5XFAD mice and non-invasive light flicker to treat the visual cortex (VC) in multiple mouse models, they found that 40-Hz gamma oscillation, but not other frequencies, reduced levels of AD pathology. Specifically, Aβ1–40 and Aβ1–42 isoforms in multiple AD mouse models (even in wild-type mice), tau phosphorylation staining in the tau P301S mouse model, and even plaque load in aged mice are reduced. Microglial responses were recruited, which is thought to be a protective function to reduce Aβ by phagocytosis. The exact manner and consequences of microglial gamma oscillation changes remain to be determined. Nonetheless, these data suggest that reducing neuroinflammation may play an important role in improving neurodegeneration.

AD affects multiple brain regions critical to learning and memory, such as the hippocampus (HPC) and medial prefrontal cortex (mPFC). Therefore, researchers speculate that gamma oscillators, being generated locally and exhibiting similar frequencies in different brain regions, can become coupled by anatomical connections between the regions and may be more conducive to alleviating AD pathology and memory loss. Recently, new research by Martorell suggested that auditory tone stimulation has similar effects to visual stimulation in increasing Aβ uptake by microglia, vascular-dilation response, and potential amyloid transvascular transport and in improving spatial and recognition memory (89) (Figure 2). More importantly, auditory stimulation combined with light-induced gamma oscillations in the hippocampal CA1 and auditory cortex regions of the brain in animal models of AD has unique effects: reducing the amyloid load across the cortex, suggesting the possibility of AD-like pathology across larger networks. Long-term treatment may be more effective than short-term treatment, by transforming neurons into less degraded states, improving synaptic function, enhancing neuroprotective factors, reducing DNA damage in neurons, and reducing the inflammatory response of microglia (90).

However, it is still unclear how non-invasive sensory stimuli are associated with endogenous gamma. Researchers have attempted to use a low-dose GABA_A_ antagonist on 5XFAD mice and found that the effects of 40-Hz flicker on Aβ levels were completely abrogated, indicating that this process may involve the participation of GABAergic neurotransmission. In addition, whether the benefits can be transformed to humans is also a crucial issue. However, manipulating neural network oscillation disturbances may be a promising strategy to alleviate pathological changes and behavioral deficits associated with neurological diseases.

### Aβ and Tau Prions Spread Through the Brains of AD Patients

Prion protein (PrPSc) is a special conformation of a protein encoded by the host, with self-reproduction ability, superior infectivity, tenacious viability, and the ability to remain concealed, even surviving in the normal denaturing environment of the digestive system. Prions can cause a variety of neurodegenerative diseases in humans, including Creutzfeldt–Jakob disease (CJD), Gerstmann–Sträussler–Scheinker syndrome (GSS), and fatal familial insomnia (FFI) in humans (91). These diseases can occur spontaneously or through genetics or infection.

Studies have shown that Aβ spreads through the brain via a pathogenic conformation similar to PrPSc (92). Brain-derived Aβ and synthetic Aβ from AD patients injected into the brain of transgenic mice showed prion-like appearances (93), which induced plaque formation and extensive deposition of Aβ. Brain extracts from age-matched patients without AD showed minimal accumulation of Aβ (94). An autopsy in a few studies also revealed that some patients had a large amount of Aβ deposition in the brain after death, after receiving *dura mater* transplantation and cadaveric growth hormone, which may mean that Aβ can be transmitted interpersonally through iatrogenic methods. Prion-like Aβ activity participates in the pathogenesis of AD. The formation of prion-like Aβ begins in one or more brain regions and then spreads to other brain regions, reflecting cross-synaptic transmission.

Tau protein is also transmitted in the brain in a prion-like manner. Previous studies have focused solely on total insoluble tau because the abundance of NFT correlates with the extent of brain atrophy and cognitive decline in AD (95). A recent study found that low prion-like tau activity is associated with longer life spans: 100 postmortem brain tissue samples from patients who died of either sporadic or inherited AD showed the presence of both prion-like Aβ and prion-like tau proteins (96). The activity of tau prion was inversely proportional to age, which means compared with the AD patients with the greatest longevity, patients who died at younger ages due to AD had lower concentrations of both prion-like Aβ and prion-like tau at the time of death, although NFT increased. This decrease in tau prion-like activity is concurrent with a decrease in tau phosphorylation, suggesting that biochemical events such as phosphorylation may affect prion-like tau formation or regulate tau toxicity, although whether the reduction of prion-like tau in elderly AD patients is due to the conversion of prion-like tau to a more inert amyloid state, such as total insoluble tau, or due to its reduced production and clearance is still unclear. Therefore, the goal of developing a therapeutic approach of AD for prions remains yet to be achieved.

### Aβ Interact With Hippocampal Ghrelin/GHSR1α Signaling in AD

GHSR1α, a member of the class A G protein-coupled receptor (GPCR) family (also known as ghrelin receptor), plays a special role in the hippocampus (97). In the healthy hippocampus, the ghrelin/GHSR1α signal affects the learning, motivational, and hedonic components of eating (98). Moreover, GHSR1α plays a role in hippocampal synaptic physiology and memory maintenance by regulating the dopamine receptor D1 (DRD1) to activate Ca^2+^/calmodulin-dependent protein kinase II (CaMKII) via the non-canonical Gαq-Ca^2+^ signaling pathway (97, 99). Hippocampal lesions are one of the earliest lesions to appear in AD and affect cognitive function (100), which may be related to GHSR1α.

**Figure 3:**
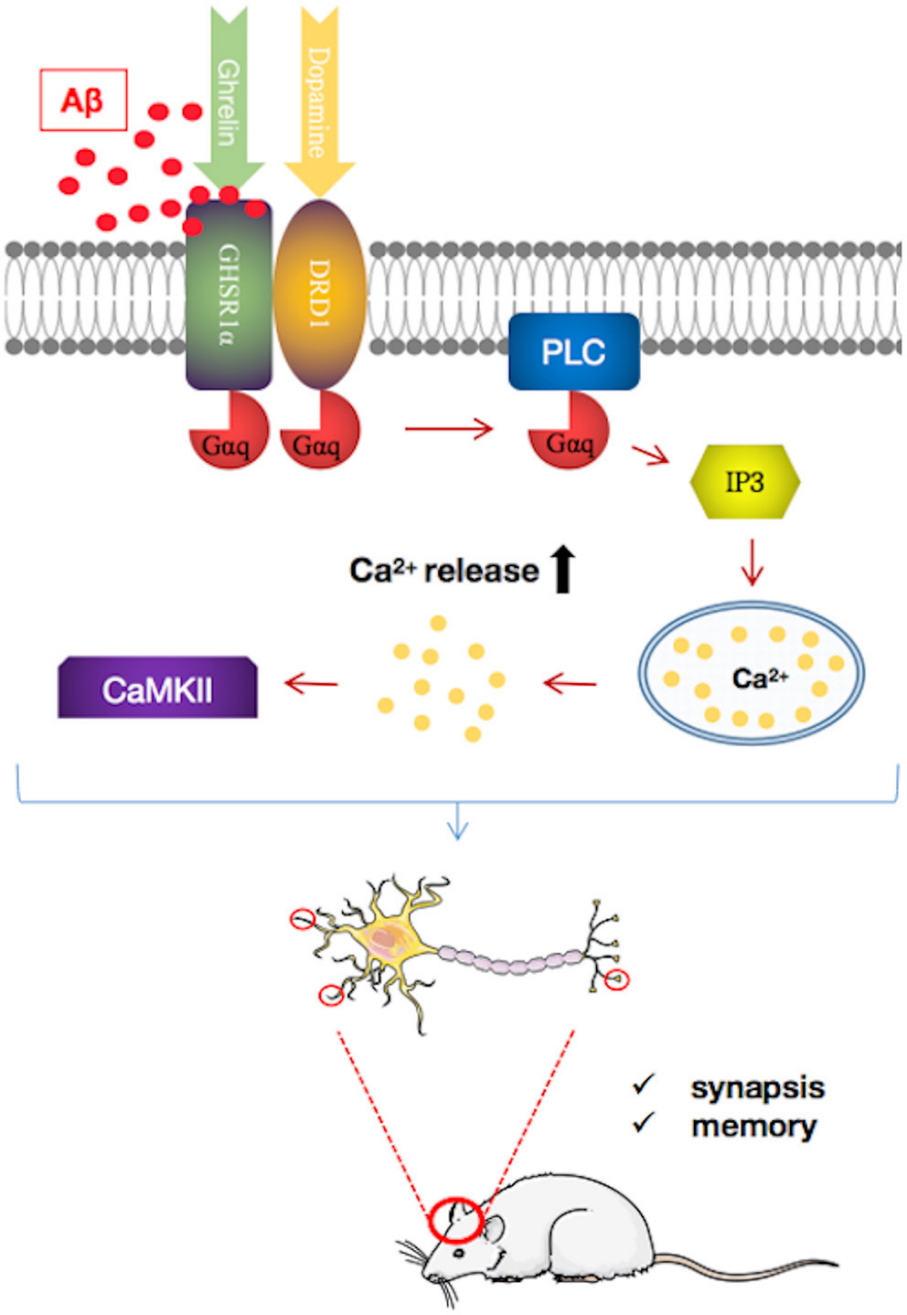
Pathways regulating growth hormone secretagogue receptor 1α (GHSR1α)/dopamine receptor D1 (DRD1) interaction by amyloid beta (Aβ) in the hippocampus of patients with Alzheimer's disease (AD). Aβ binds directly to GHSR1α and inhibits the activation of GHSR1α and prevented GHSR1α/DRD1 heterodimerization, resulting in synaptic plasticity damage and memory loss. In a mouse model of AD, simultaneous use of the selective GHSR1α agonist MK0677 and the selective DRD1 agonist SKF81297 rescued GHSR1α function from Aβ inhibition, thereby reducing hippocampal synaptic damage and improving spatial memory.

Emerging evidence suggests that the loss of GHSR1α induces AD-like hippocampal synaptic stress and memory deficits (101) (Figure 3). However, the expression of GHSR1α is increased in the hippocampus of AD patients, which may be a compensatory response to the toxic effects of Aβ. Most importantly, it is been proven that Aβ combines with GHSR1α, preventing activation of GHSR1α and GHSR1α/DRD1 heterodimerization. The resultant reduced GHSR1α/DRD1 interaction contributes to hippocampal synaptic injury, leading to memory damage (101).

GHSR1α may be a target for AD treatment. GHSR1α agonists such as MK0677 and LY444711 showed protective effects in animal and cell models (102, 103). However, clinical trials of MK0677 in AD patients failed to show clinical benefit. These results may reflect the insensitivity of GHSR1α to activators in AD patients. Furthermore, it has been shown that the combined activation of GHSR1α and DRD1 with their selective agonists MK0677 and SKF81297, respectively, rescues hippocampal synaptic function and cognition from Aβ toxicity in young 5XFAD mice (101). However, whether MK0677/SKF81297 is beneficial for older 5XFAD mice requires further investigation. Nonetheless, this study shows the potential protective effect of this dual agonist intervention in AD.

However, the roles of GHSR1α may not be limited to the above. Its role in attenuating hippocampal pathology in 5XFAD mice by neurogenesis is not to be ignored (104). Ghrelin has been shown to attenuate hippocampal pathology in 5XFAD mice by neurogenesis. Similarly, coactivation of GHSR1α and DRD1 promotes neurogenesis in the dentate gyrus of 5XFAD mice. GHSR1α dysregulation also has a huge impact on hippocampal metabolic processes and calcium signaling, which is closely related to synaptic activity and hippocampal-dependent memory impairment in elderly and AD patients (105). In addition, GHSR1α can affect the hypothalamic function and may indirectly drive hippocampal damage (106). In conclusion, the role of GHSR1α in AD cannot be ignored and may provide some promising therapeutic targets.

### Aβ Constrict Cerebral Capillaries in AD Pathology

Cerebrovascular disease can lead to changes in the function of the brain (107). In early AD, angiogenesis damage and cerebral blood flow decrease, which are thought to be the first changes of AD (108). Studies have shown that capillaries contract abnormally in brains of AD patients and that gray blood flow can reduce by about 42%. Animal studies have also found that exogenous Aβ can reduce cerebral blood flow in rats (109), which in turn promotes Aβ production (110).

**Figure 4:**
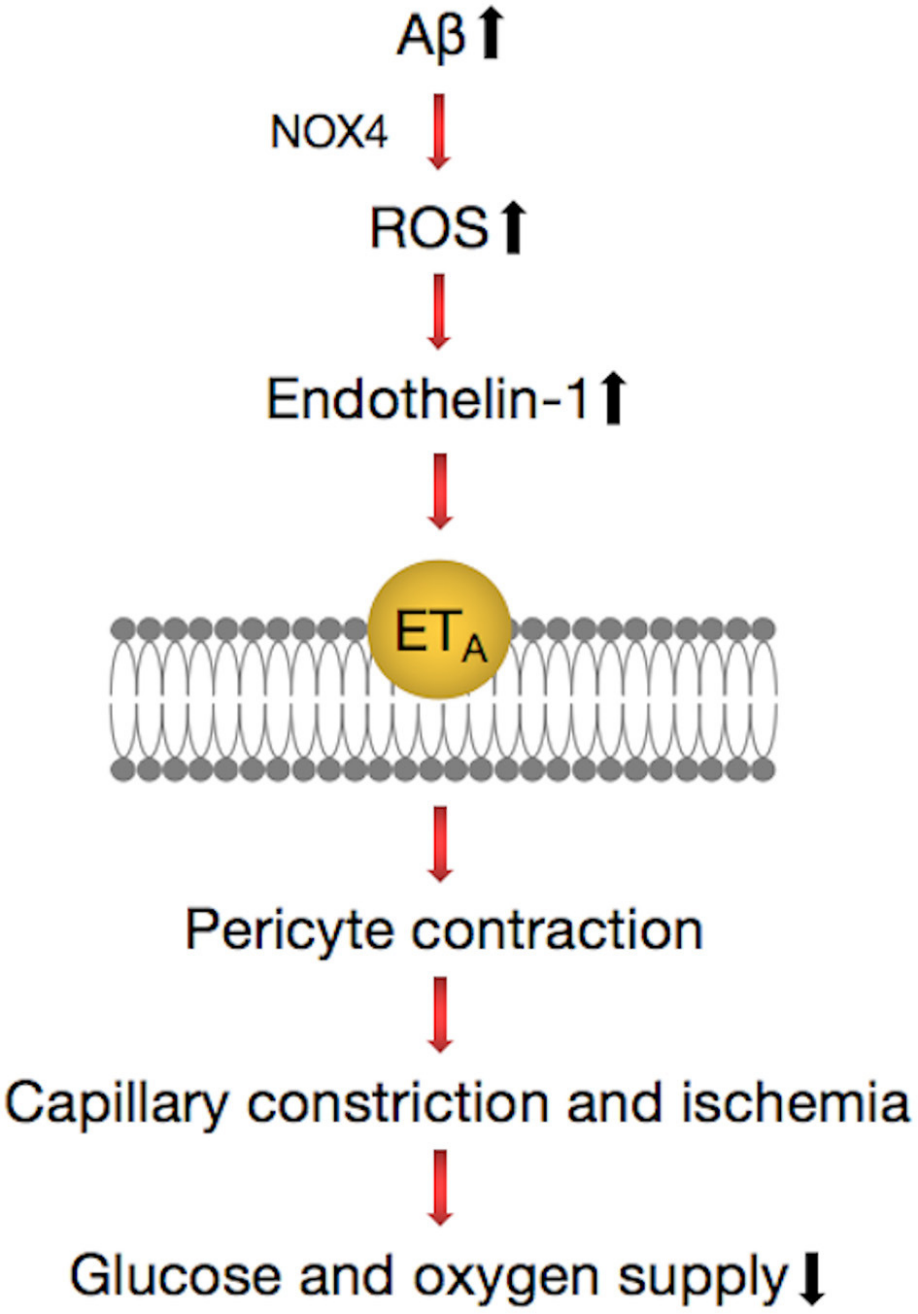
Pathways regulating contractile process of a pericyte around a capillary in the brain. Amyloid beta (Aβ) generates reactive oxygen species (ROS) (via NOX4), which evoke the release of endothelin (ET)-1, which can activate contraction by binding to ETA. These lead to the release of Ca2^+^, which evokes contraction of pericytes and brain capillaries, which leads to a decrease of the glucose and oxygen supply to the brain tissue. This pathway can be inhibited by blocking NOX4 with GKT137831 (GKT), blocking ETA receptors with BQ-123, and blocking the ET-evoked contraction by C-type natriuretic peptide (CNP).

Recently, a study published in the journal *Science* demonstrated that in the brains of AD patients, Aβ deposition shrank the blood vessels of the brain by about 8.1% and reduced the energy supply, which resulted in a decrease of blood flow of ~50%, which is close to the 42% drop in blood flow in the gray matter of AD patients (111). Aβ involved the generation of reactive oxygen species (ROS), mainly by NOX4 (reduced nicotinamide adenine dinucleotide phosphate oxidase 4), which then triggered the release of endothelin (ET)-1, thereby acting on ETA receptors to evoke pericyte contraction (111). Pericytes became stiff and necrotic after contracting, causing capillary persistent constriction and ischemia (Figure 4). The Aβ oligomer played an important role in this process. Experiments conducted on human brain slices made from normal tissue showed that capillaries in brain tissues began to contract after exposure to the Aβ composition of oligomers. No similar phenomenon occurred when Aβ monomers were used.

The vasoconstriction mechanism described suggests that there are some potential therapies for the early treatment of AD, such as the NOX4 inhibitor GKT137831 and the vasodilator C-type natriuretic peptide (CNP) (111), which can prevent the contraction of blood vessels. This implies that attention should be given to signaling pathways that act directly on neurons and suggest novel therapeutic approaches for early intervention in AD by targeting drugs to brain pericytes.

### Infection Mechanism in AD

The brain tissue of AD patients exhibits inflammation, such as the activation of complement system factors and microglia (112). The major pathological protein of AD, Aβ, has been shown to be an antimicrobial peptide, which surrounds invaders in the brain and forms plaques to protect the brain from further damage. Researchers suspected that microbes are involved in the pathogenesis of AD since 1952 (113). Herpes virus is one of the potential pathogenic factors of AD. HHV-6A, in particular, is involved in the host regulation of many AD risk genes such as BACE1 and APBB2 and promotes Aβ precipitation and neuronal loss by inhibiting miR-155 (114). *Candida albicans*, the pathogen of oral ulcers, was reported to result in a gelatinous granuloma (FIGG), similar to AD plaques and primary symptoms of suspected AD, such as memory loss (115).

**Figure 5:**
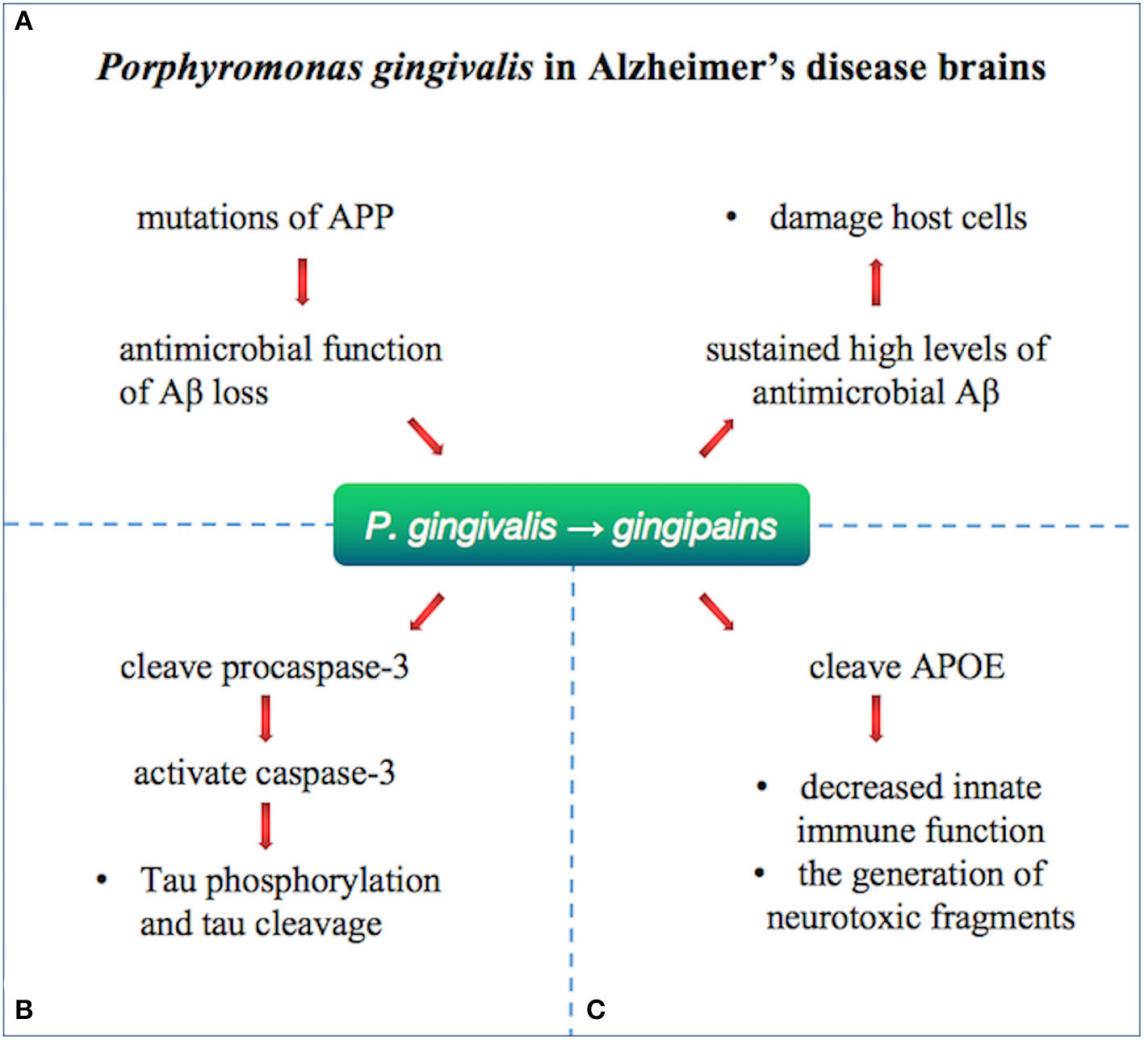
*Porphyromonas gingivalis* in Alzheimer's disease brains. *Porphyromonas gingivalis* were identified in the brain of Alzheimer's disease patients and relate to Aβ, tau, and APOE by gingipains. **(A)** Loss of biological function of Aβ as an antimicrobial peptide after APP mutation may lead to further infections. The infection with *P. gingivalis* results in brain infection of mice and induction of A1-42, which is toxic to host cells. **(B)** Gingipain proteolysis cause direct damage or activation of procaspase-3 (which can be cleaved to activate caspase-3), resulting in Tau phosphorylation and tau cleavage change. **(C)** APOE4 may be more vulnerable to gingipain cracking, producing neurotoxic APOE fragments and resulting in decreased innate immune function.

*Porphyromonas gingivalis*, the pathogen of chronic periodontitis (CP), was identified as a risk factor for dementia and AD and caused transient bacteremia through the mouth into the blood and then colonized in the organ (116). Gingipain, a cysteine protease consisting of lysine-gingipain (Kgp), arginine-gingipain A (RgpA), and arginine-gingipain B (RgpB), is considered a major virulence factor of *P. gingivalis*. *P. gingivalis* has been identified as an important risk factor for the development of Aβ plaque and AD (Figure 5). A prospective study supported that cognitive function in patients with active CP was significantly reduced within 6 months compared with AD patients without active CP (117). Infection with *P. gingivalis* in the oral cavity of mice leads to activation of the complement pathway in the brain (118). *Porphyromonas gingivalis* lipopolysaccharide has been detected in the human AD brain, suggesting its role in AD (119).

Emerging study confirmed the pathological role of *P. gingivalis* and gingipain in AD. The gingipain immunoreactivity (IR) in AD brain was significantly higher than that in non-AD control individuals. *Porphyromonas gingivalis* is reported to be present in the brain and the CSF of AD patients, indicating that the DNA of *P. gingivalis* in CSF can be used as a differential diagnostic marker. Moreover, gingipain appears in the AD brain.

Oral infection with *P. gingivalis* results in brain infection of mice and induction of Aβ1–42, which is toxic to host cells. Loss of biological function of Aβ as an antimicrobial peptide after APP mutation may lead to further infections (120). Tau phosphorylation and tau cleavage are reported to change by direct damage or activation of procaspase-3 (which can be cleaved to activate caspase-3) by gingipain proteolysis (121). *Porphyromonas gingivalis* has been hypothesized to relate to human APOE (119), producing neurotoxic APOE fragments and resulting in decreased innate immune function. Effective, selective, permeable brain small molecule gingipain inhibitors are neuroprotective (119). *In vivo*, it was shown that oral administration of small molecule gingipain inhibitors blocked gingipain-induced neurodegeneration and significantly reduced the load of *P. gingivalis* in mouse brain.

## Conclusion

The Aβ cascade hypothesis and tau hyperphosphorylation hypothesis were formulated on the basis of strong genetic, biochemical, and histopathological evidence and were later strengthened by longitudinal biomarker, cognitive, and clinical studies. However, the corresponding clinical drug research has not identified ideal therapeutics. For example, drugs that inhibit Aβ production or accelerate Aβ clearance are highly anticipated. These have probably not yet been identified because whether Aβ accumulation is the cause, or the result, remains unknown. In the meantime, many researchers have begun to explore the pathological mechanisms of AD from different perspectives. The gamma oscillations caused by auditory and visual stimulation are of note. In addition, there are Aβ and tau prion transmission mechanisms, cerebral vasoconstriction, GHSR1α-mediated mechanisms, and infection. All of these offer new strategies for AD research, and based on these, prevention and earlier treatment of AD may be possible.

## Author Contributions

LF, CM, and XH conceived and wrote the manuscript. YX and CS provided funding. SZ, ZY, ZH, HS, YF, YD, and JY collected resources.

### Conflict of Interest

The authors declare that the research was conducted in the absence of any commercial or financial relationships that could be construed as a potential conflict of interest.
